# A signaling visualization toolkit to support rational design of combination therapies and biomarker discovery: SiViT

**DOI:** 10.18632/oncotarget.8747

**Published:** 2016-05-18

**Authors:** James L. Bown, Mark Shovman, Paul Robertson, Andrei Boiko, Alexey Goltsov, Peter Mullen, David J. Harrison

**Affiliations:** ^1^ School of Science, Engineering and Technology, Abertay University, Dundee, DD1 1HG, UK; ^2^ School of Arts, Media and Computer Games, Abertay University, Dundee, DD1 1HG, UK; ^3^ School of Medicine, University of St Andrews, St Andrews, KY16 9TF, UK; ^4^ Yahoo Labs, Haifa, 31905, Israel

**Keywords:** interactive visualization, systems biology, signaling networks, combination therapy, biomarker discovery

## Abstract

Targeted cancer therapy aims to disrupt aberrant cellular signalling pathways. Biomarkers are surrogates of pathway state, but there is limited success in translating candidate biomarkers to clinical practice due to the intrinsic complexity of pathway networks. Systems biology approaches afford better understanding of complex, dynamical interactions in signalling pathways targeted by anticancer drugs. However, adoption of dynamical modelling by clinicians and biologists is impeded by model inaccessibility. Drawing on computer games technology, we present a novel visualization toolkit, SiViT, that converts systems biology models of cancer cell signalling into interactive simulations that can be used without specialist computational expertise. SiViT allows clinicians and biologists to directly introduce for example loss of function mutations and specific inhibitors. SiViT animates the effects of these introductions on pathway dynamics, suggesting further experiments and assessing candidate biomarker effectiveness. In a systems biology model of Her2 signalling we experimentally validated predictions using SiViT, revealing the dynamics of biomarkers of drug resistance and highlighting the role of pathway crosstalk. No model is ever complete: the iteration of real data and simulation facilitates continued evolution of more accurate, useful models. SiViT will make accessible libraries of models to support preclinical research, combinatorial strategy design and biomarker discovery.

## INTRODUCTION

Targeted cancer therapy aims to disrupt aberrant cellular signaling pathways. Drug targets are identified within those pathways that should be functionally linked to disease progression and have a disease specific biomarker to predict or assess therapeutic response [1]. Such biomarkers are thus surrogates of pathway state, but there has been limited success in translating candidate biomarkers to clinical practice [2]. Indeed only a tiny fraction of identified potential biomarkers have been adopted into clinical practice [3]. A key limitation to clinical translation of biomarkers is rooted in a drug design process typically framed in a single-target-single-drug paradigm [4] in the face of three major complexities: (1) the intrinsic complexity of pathway networks, (2) unforeseen feedback effects, and (3) dynamical adaptive changes in pathways when challenged by a drug.

Each of these complexities attracts different challenges. Topological complexity is an essential regulatory characteristic of cellular signaling pathways, with signaling networks exhibiting such features as pathway cross-inhibition, cross-activation, redundancy and convergence [5]. Targeted therapies can impact these regulatory processes resulting in system-scale changes to behavior reaching beyond the targeted region in ways that are difficult to predict [6]. Feedback in signaling networks likewise has a regulatory role, exerting either positive or negative effects on cascade components [7]. Feedback loops provide plasticity in signaling pathway behavior that can enable cells to adapt to therapeutic insult [8]. Further, there is a growing body of research to suggest that the strengths of these regulatory mechanisms are not static: they are dynamic over time in response to drug action [9] and so it is likely that dynamic features of network signaling might form the basis of drug targets rather than the network components themselves [10]. These complexities and dynamical adaptive changes may confer resistance to drug therapy [11] and increasing evidence from clinical studies that combination therapies offer a possible route to address drug resistance [12, 13] and cell line studies point towards the use of combination therapy to sensitize cells to anti-cancer therapy [14].

In the face of this complexity, our representations of signaling pathways and drug combinations have become increasingly sophisticated, and there is a growing opportunity for systems biology modeling to contribute to experimental design and to unravel the mechanisms and complexities of network functioning and combination therapy design [15]. For example, in a recent theoretical study of mono- and combination therapy to overcome drug resistance to kinase inhibitors [16] based on thermodynamic factors, the developed model is able to demonstrate both resistance to single drug treatment for two inhibitors when applied individually to the same kinase target and the overcoming of that resistance when those inhibitors are applied in combination. Further, through systematic investigation into model dynamics a suggested mechanism of action is identified, whereby the binding of one promoter to an inhibitor introduces conformational change in another promoter and this change provides a target for a second inhibitor to act in combination.

The value of this and other systems biology models depends on sophisticated model analysis and interpretation of model complexities, including for example the determination of system-scale control parameters such as the transition from drug resistance to sensitivity [17] and state space search optimization methods for model parameter estimation [18]. Analytical methods such as these are typically the working arena of theoreticians. Importantly, biologists and clinicians with the pertinent domain expertise are then dependent on such theoreticians to explore model dynamics, and this is a relative barrier to effective implementation of systems biology models into preclinical and clinical stages of drug development.

To overcome this barrier, we present a new, interactive, visualization and animation technology, SiViT (Signaling Visualization Tool), to enable biologists and clinicians to work directly with the model. SiViT allows biologists and clinicians to directly introduce and visualize the effects of changes in pathway dynamics *in silico* (for example by introducing mutations or inhibitors) thereby identifying unforeseen interactions, suggesting further experiments where the model is incomplete and identifying and assessing the possible effectiveness of candidate biomarkers as read outs of pathway status after dynamical adaptation. SiViT is generalizable and accessible, thus supporting preclinical research, combinatorial strategy design and biomarker discovery.

SiViT provides a single framework within which models may be imported (in Systems Biology Markup Language (SBML) format [19]) and their dynamics animated. Model structure is automatically projected onto a graph, with graph nodes representing entities in the network, such as molecular species and drugs, and edges representing node interconnections–the pathways. SiViT allows interactive animation of both species concentrations and signaling activity over time. These core features enable life scientists to animate and probe the dynamics of a cellular signaling model. Most importantly, SiViT allows domain experts to interact with the model, be it by introducing species mutations and/ or by adding specified (combinations of) concentrations of drugs at specific times.

Beyond these features, SiViT facilitates comparison of model dynamics in two different experimental regimes, for example with and without drug intervention and/ or under species mutation, through an easy to use menu system. Comparisons between experimental regimes are depicted using intuitive, color-coded animations. The result is an interactive *in silico* exploration and discovery platform to enable the life scientist to explore and exploit existing SBML-format models of cellular signaling and drug action. We illustrate these model features using as an exemplar the cell signaling model and experimental regimes described in detail in [20] and [21]. SiViT and supporting documentation is available from the authors on request together with the exemplar signaling model. SiViT requires a full installation of MATLAB 2011b (www.mathworks.com) but will automatically install all other required software.

## RESULTS

### Visualizing signaling networks with SiViT

**Figure 1:**
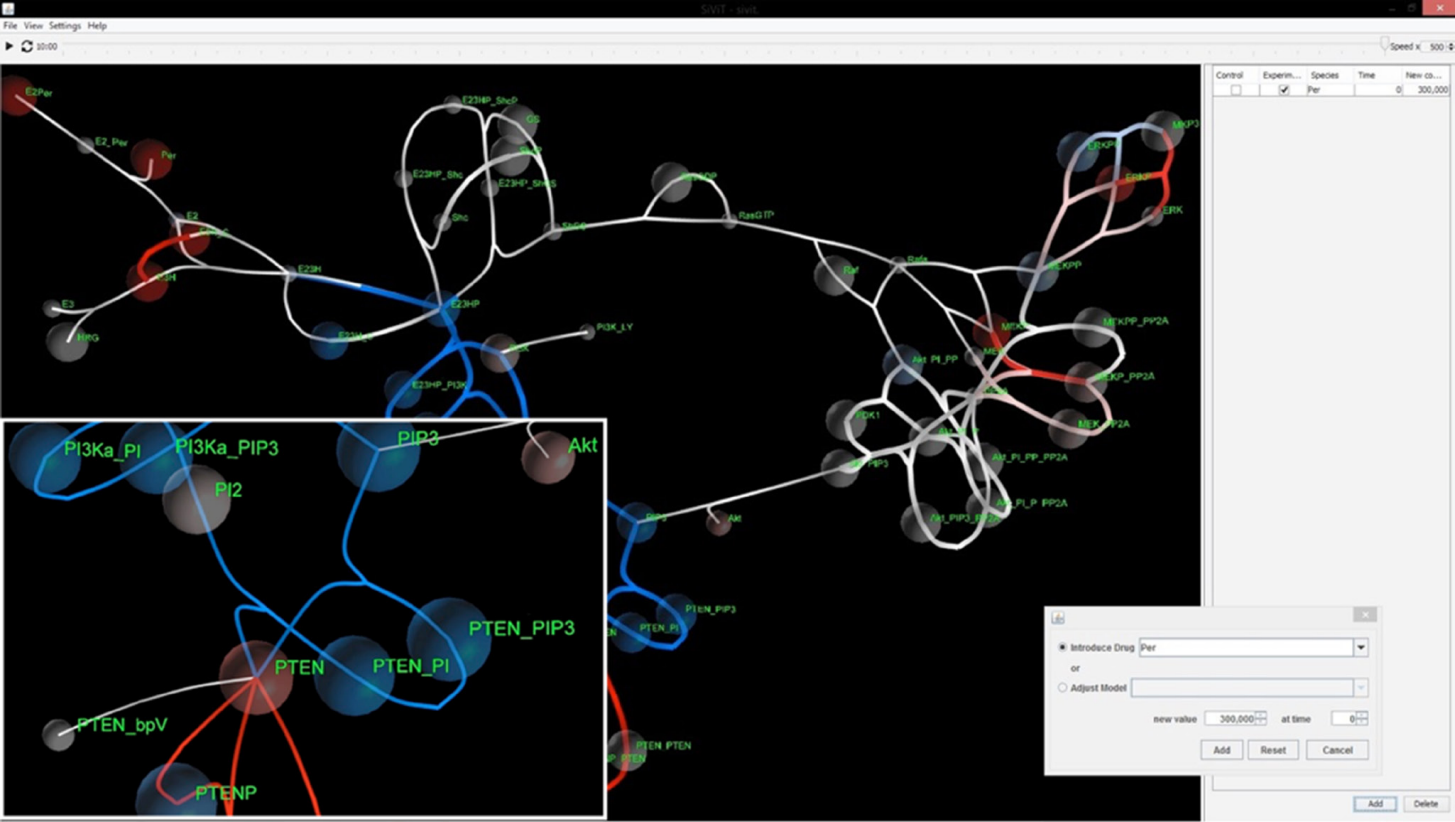
SiViT graphical user interface components Signaling network visualization with major components of a menu bar (upper top), time slider bar (lower top), central viewing frame (main area) and intervention panel (right). The visualization illustrates a pop-up dialogue box (Inset 1, bottom right) for amending drug regime and mutational status together with Inset with magnified detail (Inset 2, bottom left).

The visualization created by SiViT is encapsulated in a User Interface (Figure 1). SiViT automatically arranges the model as a network where nodes are species and edges are reactions, arranged according to a force-directed graph algorithm [22] that optimizes layout. A play icon allows visualization of model dynamics and is linked to a slider bar that allows the user to manipulate the visualization forwards and backwards in time.

The main window (Figure 1) depicts the signaling network in response to a particular *in silico* experimental regime (drug interventions, mutational status). The color scheme depends on the configuration of SiViT. For a single experiment SiViT shows the network in white. Each species is shown as a node and species concentration is depicted by the radius of a translucent sphere around that node (see Figure 1, Inset 2). Importantly this radius will increase and/ or decrease over the course of the simulation according to the calculated concentrations from within the signaling model. Reaction velocities among nodes are visualized in a similar manner: edge thickness is a function of reaction velocities and so increases and/ or decreases in line with model dynamics.

Where two different experimental regimes are set up for comparison, the visualization is tri-colored (white, red, blue). Regimes are defined via the intervention panel, and one regime is designated “Control”; the other “Experiment”. For every time point the values of each node and each edge in the Control and Experiment are compared. If there is no difference between these values the node/ edge is white; if the Experiment value is higher or lower than the Control value the node/ edge is colored red or blue respectively, with intensity proportional to this difference. The intervention panel (Figure 1, Inset 1) is a pop-up dialog box that allows the introduction of known drugs and/ or mutations. Drugs may be selected from a drop-down list and both the dosage and timing of application of that drug specified. For protein expression and catalytic activity changes indicative of mutations, any species in the model may be selected from a drop-down list and the protein concentration level or kinetic constant and the time of change in that concentration level or kinetic constant may be specified. In this way, complex regimes with multiple drugs and multiple mutations may be specified.

Additionally, and to illustrate the visualization of other models with SiViT, we loaded the SBML model of ERK signaling from [23]. In an exploration of the link between cell fate and signaling dynamics model results show that an increasing in one ERK isoform results in a decrease in the other isoform. To demonstrate model functioning we reproduced some key findings (see Supplementary Figure S1).

### Interactive animation of signaling responses to combination therapies

Using the model of the PI3K/PTEN/AKT and RAF/MEK/ERK signaling pathways developed in [20, 21, 17], we use SiViT to reveal the dynamic signaling response to: (1) application of a growth inhibitor drug; (2) introduction of a mutation in the network that is known to confer drug resistance; (3) addition of a second drug to restore network sensitivity, i.e. a combination therapy to overcome drug resistance.

**Figure 2:**
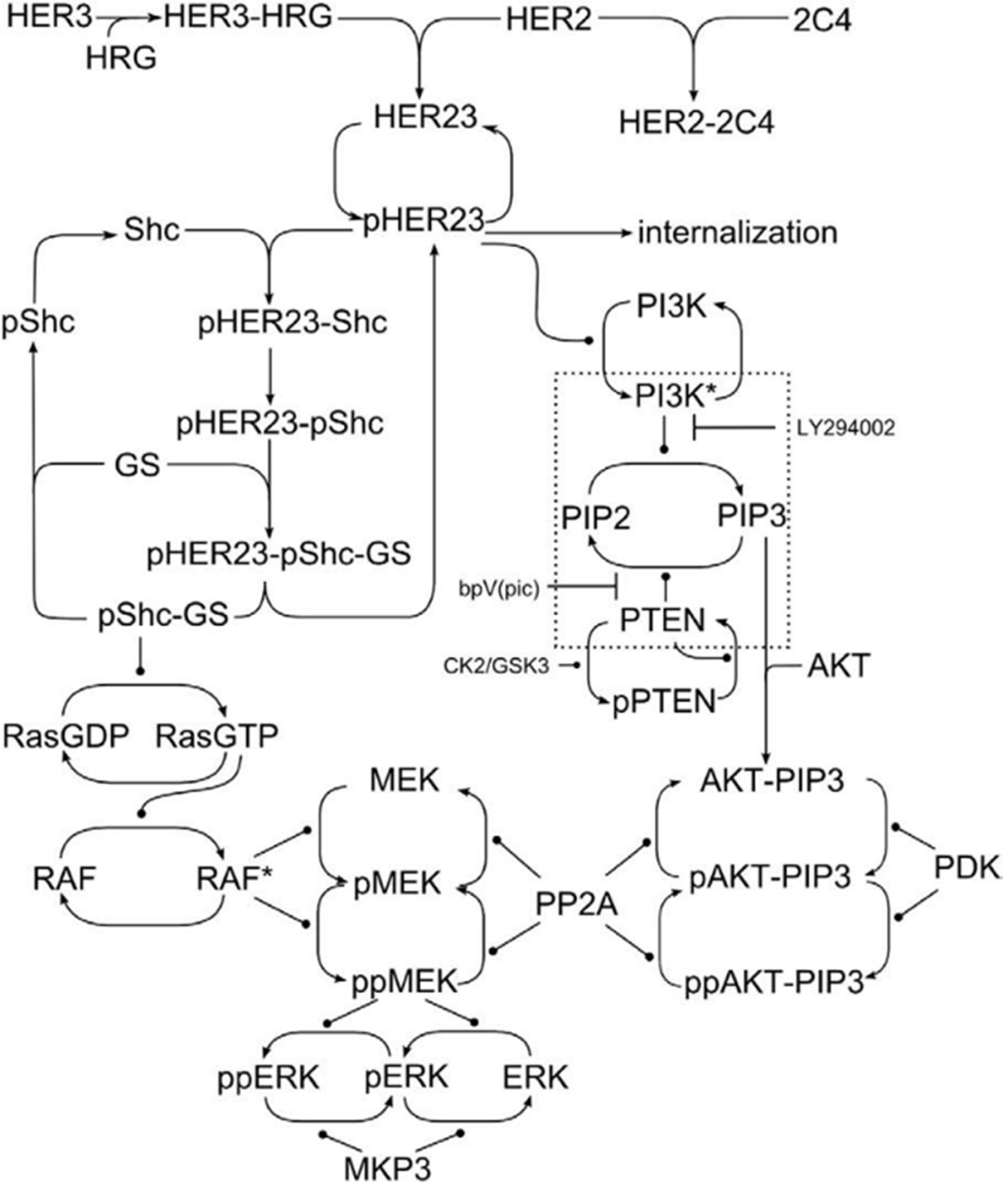
Network schematic The model of the PI3K/PTEN/AKT and RAF/MEK/ERK signaling network; figure reproduced from [17].

**Figure 3:**
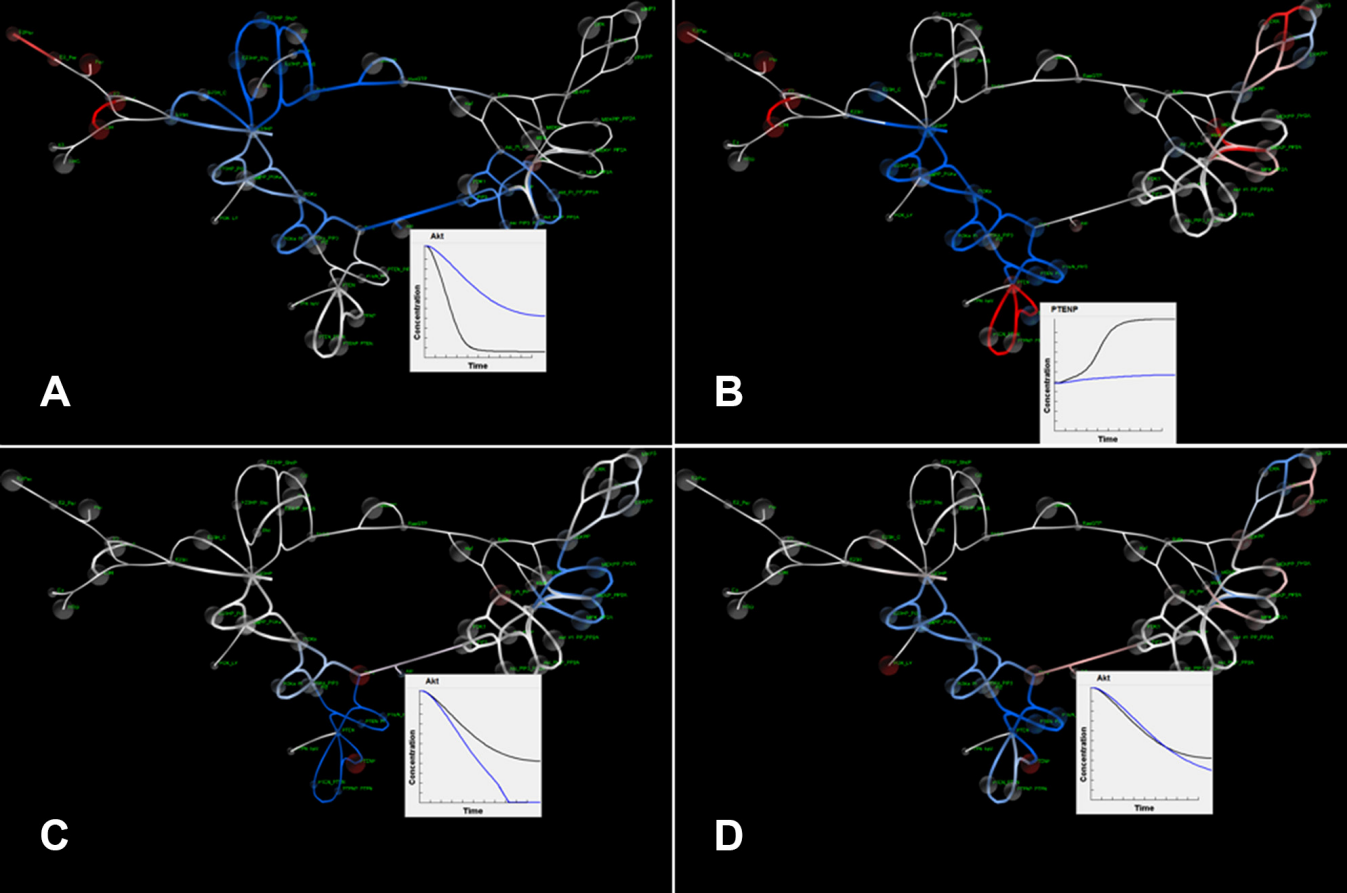
SiViT visualizations of PI3K/PTEN/AKT signaling (**A**) PI3K/PTEN/AKT signaling network showing substantial down-regulation in blue. Inset shows increase in AKT levels after 1 minute following pertuzumab application (blue line); (**B**) PI3K/PTEN/AKT signaling network 10 minutes after pertuzumab application. The network shows increases in signaling with respect to signaling at 1 minute (Figure 3A). Inset shows the relative decrease in phosphorylated PTEN level following inhibition (blue line); (**C**) The effect of PTEN loss on the efficacy of pertuzumab compared with pertuzumab action in a network with no mutation after 10 minutes. Inset shows a drop in AKT, representing increased AKT signaling following mutation (blue line); (**D**) The response of the signaling network after 10 in response to combination therapy to overcome acquired resistance: Inset shows that signaling levels in the mutated-resistant network (blue line) match those for normal functioning (black line).

The computational model describes the signaling response kinetics to heregulin, a growth factor that binds with receptors in the Erbb family to induce HER3/HER2 receptor dimerization pHER23 (see model schematic in Figure 2). This in turn stimulates tyrosine phosphorylation that in turn drives intra-cellular signaling activity. This signaling activity can be inhibited by receptor tyrosine kinase (RTK) inhibitors, and we include one such inhibitor in our model: pertuzumab (2C4 antibody). Pertuzumab is designed to target HER2 to inhibit HER2 dimerization with other Erbb family members, and especially formation of the oncogenic HER2-HER3 dimer. This HER2-HER3 dimer can activate the PI3K/PTEN/AKT signaling pathway that governs cell survival and so proliferation and tumor growth. Pertuzumab thus acts to suppress activation of the PI3K/PTEN/AKT cell survival pathway. Supplementary Videos S2 to S4 and still Figure 3A–3D show the dynamical response of this model visualized through SiViT, and show the PI3K/PTEN/AKT pathway vertically downwards.

Supplementary Video S2 shows the addition of 30 nM pertuzumab at the beginning of the simulation, and animates the network dynamics in response to drug action compared to network functioning without drug application. The drug is added through a drop-down menu of available therapies, with a standard dosage provided that can be edited, and the drug is added at the current time point in the simulation by default (also editable to allow for sequential application). The first minute of simulation dynamics shows the early network response to the addition of the drug (top left): regions depicted with higher activity are the node in the network representing the drug concentration and the HER2 node to which it binds. Additionally, increases in HER3 and HER3 bound with heregulin are observed (in red) since HER2 inhibition limits HER2/HER3 dimerization and so more HER3 is free.

SiViT highlights the impact of pertuzumab on network signaling following drug action, and shows this impact propagating along both PI3K/PTEN/AKT and RAF/MEK/ERK pathways over time in Supplementary Video S1. Figure 3A depicts the response of the signaling network to 30 nM pertuzumab after 1 minute compared with normal functioning in the absence of pertuzumab. This reduction in signaling activity was constant throughout the simulation: Figure 3A shows the simulation after 10 minutes. The key output from this pathway, AKT, is shown in the graph insert in Figure 3A: SiViT analysis revealed an increase in AKT, and so a decrease in active, phosphorylated AKT over the 10 minute simulation. Note the single node to the far left of this pathway is the input node for a second drug.

Within this pathway, the PTEN-pPTEN cycle response was time variant as shown in the Supplementary Video S2: PTEN concentration level decreased over time in normal functioning since activated PTEN, i.e. pPTEN, increased. Following inhibition by pertuzumab those species that would normally bind with PTEN were inhibited (blue) and so PTEN levels reduced at a much slower rate. Figure 3A shows no (notable) change in PTEN level after 1 minute; Figure 3B reveals the relative increase in PTEN level following inhibition and the graph insert in Figure 3B shows PTEN level over the whole simulation.

A similar time variant is observed in the RAF/MEK/ERK signaling pathway. Figure 3A–3D shows the RAF/MEK/ERK pathway across the top of the signaling network. As a consequence of the reduction in input signal pHER23 following HER2 inhibition by Pertuzumab we observed a reduction in signaling activity in the whole pathway at 1 minute (Figure 3A). At 10 minutes (Figure 3B) we observed increases in signaling activity. Importantly, and in contrast to PI3K/PTEN/AKT signaling, this represents a time lag in signaling activity. The reduced input pHER23 slowed down the rate but not the level of signaling in this pathway: at the 10-minute time point measured levels are then higher but this is an artefact of differential phasing. These dynamics can be observed in Supplementary Video S2.

Next we introduced a mutation, PTEN loss, into the network associated with resistance to anti-cancer drug therapy. PTEN loss was represented in the model by a 50% reduction in original PTEN level. Again we focused on AKT signaling and the impact of PTEN loss on the effectiveness of pertuzumab in inhibiting AKT signaling. Note that both our model and experimental systems confirmed that PTEN loss alone does not influence AKT activity. Supplementary Video S3 shows the introduction of that mutation through the Adjust Model section of the dialog box, and the resulting network dynamics in response to pertuzumab compared with a network response without this mutation. SiViT reveals an increase in AKT signaling, shown in the edges connected to the AKT node and these are visible from one and a half minutes onwards. Supplementary Video S3 also shows progressive decreases in the region surrounding PTEN (lowest part of the network in Figure 3C) manifest in the various PTEN complexes (see schematic in Figure 2), with some nodes that would otherwise interact with PTEN showing an increase due to lower levels of complex formations. Figure 3C shows the effect of PTEN loss on the efficacy of Pertuzumab compared with Pertuzumab action in a network with no mutation. Figure 3C depicts a marked decrease in the amount of AKT (see graph insert) over the 10-minute period, reflecting the increase in AKT signaling. Finally, the decrease in MEK, pMEK and ppMEK is due to cross-talk between the PI3K/PTEN/AKT and RAF/MEK/ERK signaling pathways, e.g. lower levels of activating, phosphorylated RAF.

We then restored network sensitivity to pertuzumab following PTEN loss with the addition of a second drug, the PI3K inhibitor LY294002. The combination therapy of pertuzumab and LY294002 was identified via an *in silico* perturbation analysis and subsequently confirmed via *in vitro* experiments [17], to derive a control parameter that governs the signaling response of the PI3K/PTEN/AKT pathway to pertuzumab. This control parameter encapsulates the ratio of PTEN to the product of active PI3K and AKT, and so PTEN loss can be compensated for by PI3K inhibition (or AKT inhibition). Supplementary Video S4 and Figure 3D show the response of the mutated network to this combination therapy compared to the normal network response to pertuzumab. Note that in [17] we reported on 5000 nM of LY294002; here we used SiViT to explore the parameter space to identify a very close match to AKT signaling in normal response to pertuzumab (see Inset graph) with only 100 nM of LY294002. The down-regulated region in Figure 3D at 10 minutes of simulation time occurs because of the reduction in concentrations of PTEN (through mutation) and PI3K (through drug action). Supplementary Video S4 reveals the dynamics of this down-regulation, and shows differential, increased down-regulation of the PI3K/PTEN/AKT pathway after 1 minute compared to the original sensitive network (Supplementary Video S2). This difference is short-term and after approximately 7 minutes there is no substantial difference between the original sensitive network and this network where sensitivity has been restored through combination therapy.

### Dynamics of biomarkers of drug resistance

**Figure 4:**
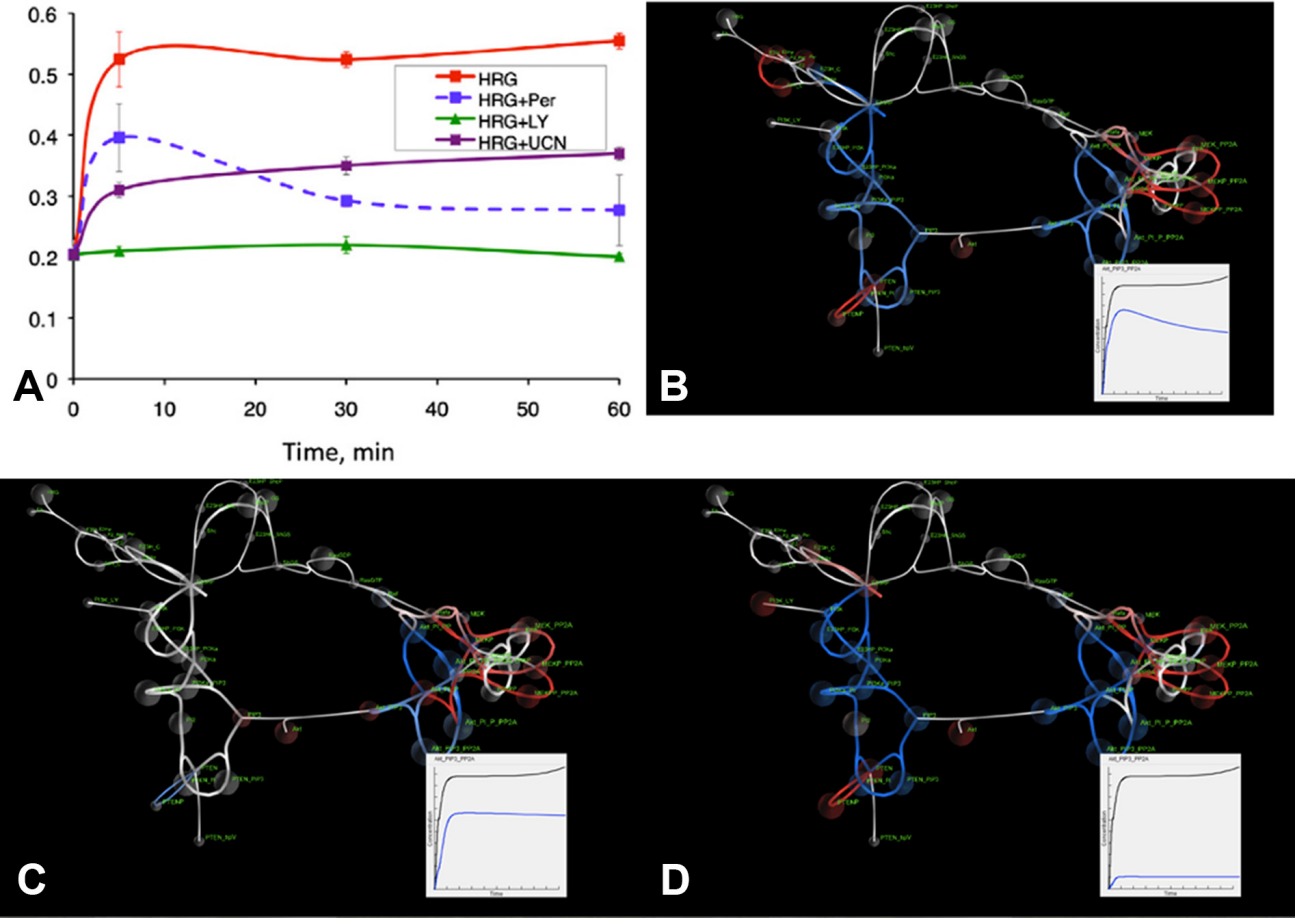
Experimental results and SiViT visualizations of signaling dynamics in the OVCAR4 cell line Figure 4A shows phosphorylated AKT over one hour in response to heregulin stimulation (red line) combined with pertuzumab (blue line), PI3K inhibitor LY294002 (green line) and PDK1 inhibitor UCN-01 (purple line); Figure 4A reproduced from [24]. Figure 4B–4D shows SiViT visualizations after one hour; Insets show integrated AKT signaling in the control condition (black line; heregulin only) and in response to pertuzumab (Figure 4B), LY294002 (Figure 4C) and UCN-01 (Figure 4D).

In [24] we used a slightly modified version of the model presented in Figure 2 and developed a global sensitivity analysis (GSA) approach to support exploration of the effect of adjusting multiple model parameters on signaling pathway activity, with a particular focus on phosphorylated AKT. Biomarkers of AKT signaling dysregulation were determined based on those parameters that had the highest levels of sensitivity for pAKT signaling levels. Figure 4A–4D show the integration of GSA and SiViT analyses for the identification and interpretation of biomarkers PDK1 and PI3K. Figure 4A shows experimental results and model predictions for phosphorylated AKT signaling dynamics in OVCAR4 cell line in response to heregulin stimulation and drugs targeting either HER2 growth receptor or the identified biomarkers PDK1 and PI3K. Figure 4B–4D shows SiViT visualizations of the OVCAR4 cell line for each experimental condition in Figure 4A.

Supplementary Video S5 and Figure 4B show the response of the signaling network to heregulin and 30nM of Pertuzumab after 60 minutes compared with normal functioning. The effect of pertuzumab is similar to the PE04 network of Figure 3A: SiViT analysis revealed an increase in AKT, and so a decrease in active, phosphorylated AKT over the simulation. Following inhibition by pertuzumab those species that would normally bind with PTEN were likewise inhibited (blue). Of note is the effect of pertuzumab on signaling downstream of AKT (far right of Figure 4B). Downstream AKT complexes were down-regulated; in contrast, SiViT revealed upregulation in the MEK signaling cascade that binds with PP2A. PP2A provides cross-talk between AKT and MEK pathways, and it is this cross-talk that drives upregulation of MEK-PP2A complexes. Down-regulation of AKT causes an increase in availability of PP2A, the levels of which remain largely constant following pertuzumab, resulting in increased MEK-PP2A complexes. The effect of this cross-talk becomes increasingly pronounced over the time course of the simulation as shown in Supplementary Video S5. The Inset in Figure 4B shows integrated pAKT signaling in response to heregulin the presence (blue line) and absence (black line) of Pertuzumab and these show good agreement with the blue (HRG+2C4) and red (HRG) lines in Figure 4A respectively.

Figure 4C and 4D show the network response to heregulin and either PDK1 inhibition with UCN-01 (Figure 4C) or PI3K inhibition with LY294002 (Figure 4D) compared to the network response to heregulin. Figure 4A shows PDK1 inhibition is less effective at reducing AKT signaling than Pertuzumab. In addition to this single measure, Figure 4C shows the effect of PDK1 inhibition on the entire network after 60 minutes, and reveals markedly less inhibition upstream of AKT compared with inhibition by Pertuzumab. Down-regulation and upregulation of the AKT and MEK signaling cascades respectively are comparable with signaling activity following pertuzumab and this is driven by the same PP2A cross-talk. Figure 4D shows the effect of PI3K inhibition on the whole network. Network response to PI3K inhibition is broadly similar to Pertuzumab inhibition across the network, although the down-regulation of the PI3K/AKT pathway is more pronounced following PI3K inhibition. Insets in Figures 4C and 4D show time course dynamics of the integrated pAKT signaling in response to heregulin the presence (blue line) and absence (black line) of PDK1 and PI3K inhibition respectively, and these show good agreement with the purple (HRG+UCN-01) and green (LY294002) lines in Figure 4A.

## DISCUSSION

We have developed an interactive animation tool that can import any suitably formatted dynamical model written in SBML. SiViT is compatible with a wide range of curated models stored on the open access EMBL-EBI BioModels database (https://www.ebi.ac.uk). We provide scripting that converts those models to a form executable by the SimBiology toolbox. Models may be uploaded to the BioModels database for curation and then used with SiViT. Additionally, our software is open source and so other researchers are able to provide bespoke conversion scripts, in either Java or MATLAB, guided by our scripts or otherwise.

SiViT allows a non-computational specialist user to interrogate the possible effects of a drug, a combination of drugs and the response of a biomarker to adaptive change in the tumor cell. Biomarker elucidation in the context of combination therapies attracts the challenge of searching through a large state space of possible drug targets, pathway status readouts, drug dosages and timings of application in a problem domain characterized by non-linearities, topological complexity and dynamic rewiring. Systems biology models can capture some of that complexity as exemplified in our consideration of signaling responses to combination therapies. Computational search frameworks can explore that state space in a focused, directed manner as exemplified in our elicitation of biomarker dynamics. SiViT affords observation of those complexities in a given network and allows easy exploration of model dynamics and sensitivities that can inform the search criteria crucial to success of any computational search framework. SiViT can make a direct contribution to complement existing efforts in this arena of study.

For example, [25] describe a model for predicting the impact of combination therapies on the RAS/PI3K signaling network for a panel of cell lines with different mutational status. They show how model analysis can support the identification of combination treatments and subsequently confirm the predictions from the model in a xenograft system. A central issue raised is that only particular combination treatments work for particular cells and modeling can guide this challenging discovery process. Complementary to such model analysis, our approach allows biologists and clinicians to design *in silico* combination treatments by adding drugs to different cell lines with specified doses and mutations through a menu interface and observing the impact on the signaling network.

Moreover, given a growing awareness that it is not simply the mix of drugs that constitute a combination therapy but also the scheduling of their application, SiViT can support sequential application studies. For example, [9] undertook a sophisticated combined experimental and theoretical study of the sequential application of anti-cancer drugs. They noted that complexities in signaling networks such as feedback and cross-talk make predicting cellular responses to drug action difficult and especially so in cancer cells since functioning is aberrant. This difficulty is compounded for combination therapies. [9] targeted triple negative breast cancers and showed that EGFR inhibition prior to DNA damaging chemotherapy (doxorubicin) sensitizes some cell lines to that damaging agent. Analysis of gene expression profiles of cell lines that were both sensitive and insensitive to time-staggered EGFR inhibition followed by doxorubicin revealed marked differences in genes including those linked to key survival and inflammation pathways. Further proteomic analyses revealed differences in pathways, including those linked to survival, in cells sensitive to sequential combination therapy. This response is explained in terms of a rewiring of the signaling pathways to sensitize cells to doxorubicin as a result of pre-treatment with EGFR inhibitor; co-treatment or post-treatment did not sensitize cells.

The notion of pre-treating cells to promote sensitivity to a second treatment seems intuitive, yet other work highlights further complexities in sequential combination therapy [26]. In this study a time-dependent effect of PI3K/mTOR inhibition on doxorubicin-induced apoptosis in neuroblastoma cells was observed. Post-treatment with the PI3K/mTOR inhibitor most sensitized the cells to doxorubicin treatment; the sensitizing effect was less pronounced in co-treatment and pre-treatment. This observation reveals that the order of application of combination therapy depends on context. SiViT provides a platform that supports such contextual investigation. Drugs can be added in any order, with each added at an individually specified time and dose. Comparison between different regimes, which could vary in timing and or dose, allows *in silico* optimization of time-staggered combination therapies.

Clearly, the identification of biomarkers and the design of effective combination therapies are challenging and require systematic experimental study informed by systems biology modeling. We propose that SiViT provides a valuable bridge between the fields of cell biology and computational modeling, enabling cell biologists and clinicians to explore available models of signaling pathways and drug actions in an environment that does not require expert computational modeling expertise, simply an awareness of the process of modeling. Through our generalizable technology we seek to promote the uptake of modeling by the biological and clinical communities in support of preclinical research, combinatorial strategy design and biomarker discovery.

## MATERIALS AND METHODS

### SiViT framework

**Figure 5:**
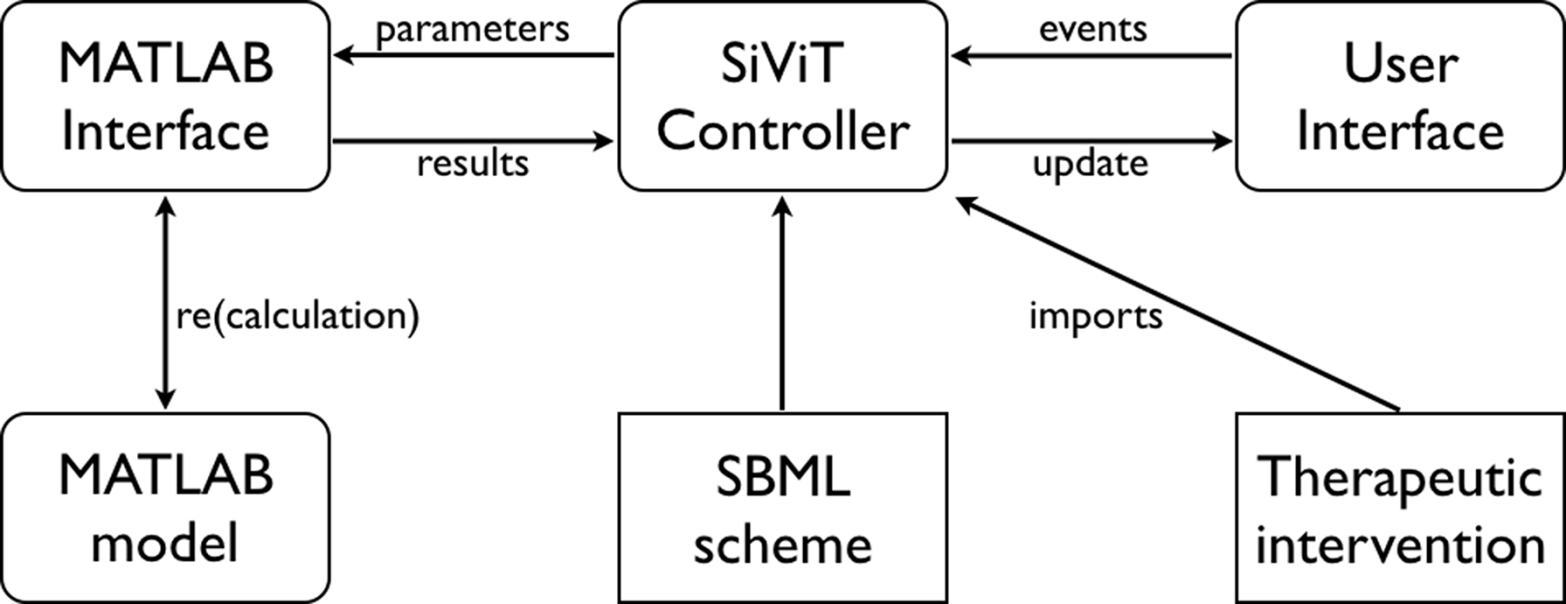
SiViT architecture Interoperation of major components (rounded rectangles), external resources (rectangles) and key interactions (arrows). Through the User Interface, users may select an SBML file containing the SBML scheme for the model, and load that model into SiViT with an accompanying set of therapeutic interventions (a file of [drug name, dosage] pairs). SiViT then constructs a model as defined by the SBML, passed as parameters to MATLAB, and captures all time series data computed by MATLAB, i.e. the results, pertaining to all biomolecular species. The visualization is based on both the model structure, i.e. species and reactions as prescribed in the SBML, and the results of model execution, i.e. species concentrations and reaction velocities over time. A user may make changes to that model via the interface, e.g. by adding a drug at a prescribe time, and this generates an event that is translated into a change in model parameters. This in turn results in a model recalculation, the results of which are passed back for re-visualization.

SiViT comprises three major components: a controller interlinking interfaces to both the user and to MATLAB for model (re)calculation, and is implemented as a suite of Java program files. Figure 5 depicts the overall structure of SiViT. SiViT requires as external files the cellular signalling model itself as implemented in MATLAB with the model structure represented within a SBML scheme, a list of therapeutic interventions (drug name and typical concentration) and an optional set of 3D graphical object files (not shown).

Central to this architecture is the SiViT controller, which has two major roles: to import both a specific signaling model and a predetermined list of model interventions; and to translate both user interactions with the visualized model into updated parameter sets for MATLAB, and model results into the visualized model. Optionally the SiViT controller can import 3D graphical objects illustrating each node (species) to enhance visual aesthetics (not shown in Figure 5; see User Interface below).

Importing the model requires the reading in of the SBML scheme that defines the model. Implemented using the matlabcontrol (code.google.com/p/matlabcontrol/) Java API to MATLAB, the SiViT controller establishes a communication protocol to MATLAB in terms of parameters and forms a software link in order to invoke the MATLAB model, managed by the MATLAB interface. The list of pre-defined therapeutic interventions is a file of [drug name, dosage] pairs; note dosage (in nM) and time of application can be modified through the User interface. Note interventions not on this list can be introduced easily. The algorithm describing SiViT operation is provided in Supplementary Text S6.

### MATLAB interface

Interlinking the MATLAB model and the User interface is more complex. The User interface drives the dynamics of this interlink. In summary, the loading of a new model and changes to a model through the User interface (see below) generate interface events that are passed to the SiViT controller and converted into changes to the parameter set for the MATLAB interface. The MATLAB model is then (re)calculated and results passed back to the SiViT controller via a data structure. This data structure is then processed and passed to the User Interface for an updated visualization. Note the User interface is able to specify and then compare two different model regimes, and in this case the SiViT controller manages two concurrent data structures: one for each set of model results.

In more detail, the MATLAB interface is provided with a data structure capturing the form of the parameter set of the signaling model in terms of names of both species and reactions together with protein concentrations and reaction velocities over time. Through the matlabcontrol API the selected signaling model is executed and the time series of results (concentrations, velocities) updated. Any interventions added to the model through the User interface are captured as user-generated events and added to the parameter set for the signaling model. When the SiViT controller detects a parameter set change the MATLAB interface triggers recalculation of the model. The computed model may then be queried through this MATLAB interface for all species and reaction names, and all protein concentrations and velocities over time. This combination of intervention addition, model recalculation and state variable query provide all the data to feed the User interface.

### User interface

The User interface is shown in Figure 1 and comprises a menu bar (upper top), time slider bar (lower top), central viewing frame (main area) and intervention panel (right). The menu bar allows the loading of a model into SiViT, and saving of the visualized model image (.jpg) at any point in the simulation. The interactive environment allows zooming in/out and rotational control of the model to support exploration and this combined with image saving, provides a means to record model dynamics.

The loaded model is arranged on screen procedurally, automatically accounting for the size and topological features of the signaling network. The view itself may be specified as 2D or 3D (allowing rotation around axes). Within this high-level constraint the spacing of the nodes is implemented as a force-directed graph layout algorithm [22]. This algorithm seeks to optimize the network structure such that edges are of equal length and that edges do not intersect (in 2D or 3D space as specified). In our implementation, and to provide additional flexibility in layout choices, it is also possible to constrain the layout algorithm such that the network is arranged onto the surface of a sphere.

The time slider bar allows the user to move forwards and backwards in time through simulation dynamics simply by moving the slider between 0 and maxTime, the maximum duration of the simulation. By pressing the play icon to the left of the slider bar, the slider icon on this bar will move from left to right automatically during the simulation. The play function can also be paused, and the slider moved by the user (forwards and backwards in time). Finally, a small dialogue box to the right of the slider bar allows modification of the speed of model animation.

The central viewing frame holds the signaling network itself, and the detail of the visualization is dependent on whether SiViT is being used to explore the dynamics of a single experimental regime or compare two different regimes. For a single experimental regime the visualization is monochrome (white). Nodes in the network depict species concentration: the radius of the sphere is proportional to the species concentration and so varies in line with model dynamics. If the optional 3D graphical objects are imported the center of the node is a bespoke volume image for that species; if not it is a yellow volume image. Reactions are shown as inter-node connections, i.e. graph edge, and edge thickness is proportional to reaction velocities and so varies in accordance with model dynamics over the course of the simulation.

## SUPPLEMENTARY MATERIALS FIGURES










