# Supplementary material for: A signaling visualization toolkit to support rational design of combination therapies and biomarker discovery: SiViT
Source: Oncotarget. 2016 May 18;8(18):29657–67. doi: 10.18632/oncotarget.8747 (PMC5444693; doi:10.18632/oncotarget.8747)
Supplement: Supplementary file 1 [file oncotarget-08-29657-s001.pdf]

A signaling visualization toolkit to support rational design of combination therapies and biomarker discovery: SiViT

Supplementary Materials

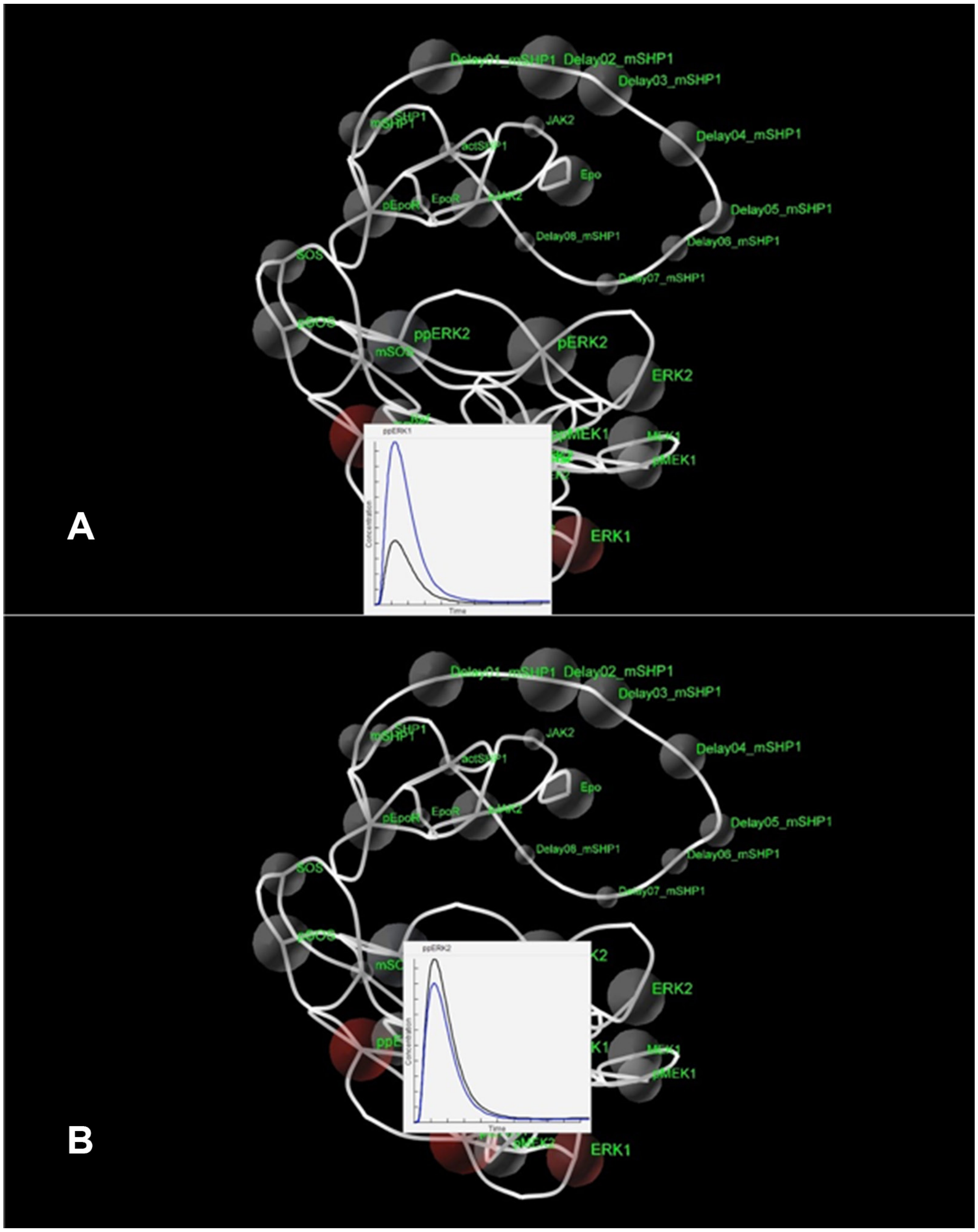

Supplementary Figure S1: SiViT visualization of the model of Schilling et al. [23]. Phosphorylation patterns of ERK1 and ERK2 following a three-fold increase in ERK1 concentration. As per Figure 4C in [23], increasing ERK1 concentration results in a marked rise in ppERK1 levels vs the control condition (Figure S1A blue and black line respectively) together with a small decrease in ppERK2 levels vs the control condition (Figure S1B blue and black line respectively).

Supplementary Materials Video S2: Shows the addition of 30 nM pertuzumab at the beginning of the simulation, and animates the network dynamics in response to drug action compared to network functioning without drug application.

Supplementary Materials Video S3: Shows the introduction of a network mutation and signaling following the addition of 30 nM pertuzumab at the beginning of the simulation.

Supplementary Materials Video S4: Shows restoration of network sensitivity to pertuzumab following PTEN loss with the addition of a second drug, the PI3K inhibitor LY294002.

Supplementary Materials Video S5: Shows the network response to 30 nM pertuzumab at the beginning of the simulation, and animates the network dynamics in response to drug action compared to network functioning without drug application over the course of 60 minutes.

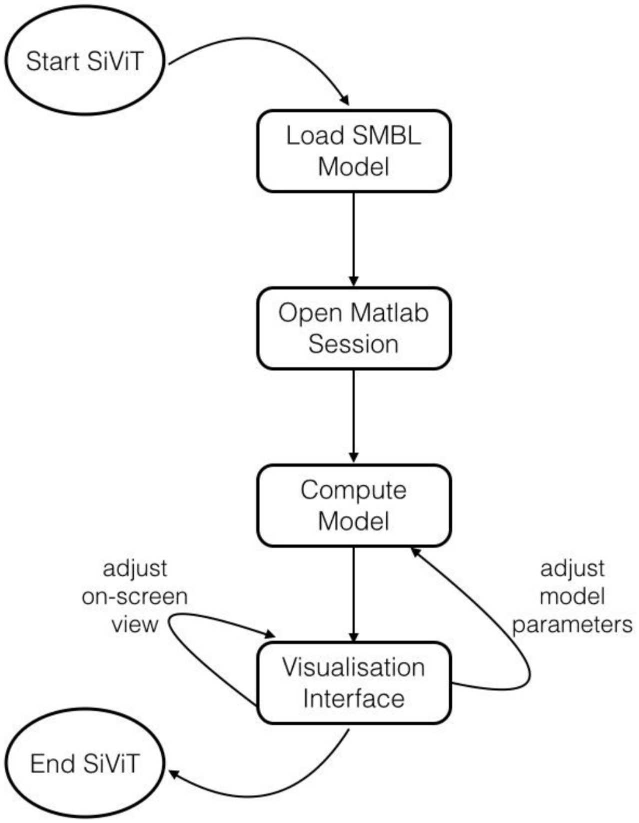

Supplementary S6: Flowchart and Pseudo-code listing for SiViT

```
/*** SiViT pseudo code ***/
/*
* This pseudo code reflects the general flow of the SiViT application.
* Only key features are highlighted in this document.
* The structure does not necessarily match that of the Java application files.
* Some function and variable names were changed to improve readability.
*/

/** Main Cycle and User Interactions **/
setup
check dependencies
GET MATLAB installation path from Windows registry
```

```
OPEN MATLAB session
GET drug settings from file
IF "File ---> Open" menu item selected
DISPLAY file selection window
SELECT model to load
LOAD user specified model file parse model file
pass model variables to MATLAB
CALL MATLAB script to simulate model

SET default simulation time to 1000 milliseconds (10 minutes)
simulate model

IF "Settings ---> Max Sim Time" button pressed
DISPLAY simulation time settings window
SET new simulation time
pass new value to MATLAB and re-simulate model
IF "Add" button pressed
add event
IF introducing drug
SELECT drug from the list
SET concentration value
SET time when changes will take effect
pass adjusted parameters to MATLAB
IF adjusting model (mutation)
SELECT model variable to adjust
SET new value
SET time when changes will take effect
pass adjusted parameters to MATLAB
re-simulate model
GET simulated data from MATLAB
construct 3D model of simulated data (see Visualisation section)
apply force graph to 3D model
DISPLAY model
IF INPUT mouse-wheel zoom camera in/out
IF INPUT mouse left-click and drag rotate camera
IF INPUT mouse middle-click pan camera
IF INPUT mouse right-click
IF click on Node OR Edge
construct line chart of concentration levels (see Line Chart section)
DISPLAY line chart
IF "Play/Pause" button pressed
play/pause/resume animation
IF "Loop" button pressed
toggle loop on/off
IF "Speed" value changed
change animation speed accordingly

/** Visualisation **/
GET simulated model data
construct visualisation objects
FOR every species
construct Node
IF model file present
LOAD model
ELSE
CREATE sphere
CREATE 2D text with species name
SET default size
SET colour to white
FOR every reaction
construct Edge
CREATE line-strip
SET default width
SET colour to white

CONFIGURE lighting
CALCULATE Force Model
SET Nodes positions
SET Edges connecting Nodes

/** Animation Cycle **/
FOR every simulated time fragment
IF using Control Run
SET Node size based on species concentration value
SET Edge thickness based on reaction speed
IF using Experiment Run
SET Node size based on new species concentration value
IF Experiment concentration < Control concentration
SET Node colour as blue
SET hue value based on difference between Experiment and Control
IF Experiment concentration > Control concentration
SET Node colour as red
SET hue value based on difference between Experiment and Control
SET Edge thickness based on new reaction speed
IF Experiment reaction speed < Control reaction speed
SET Edge colour as blue
SET hue value based on difference between Experiment and Control
IF Experiment reaction speed > Control reaction speed
SET Edge colour as red
SET hue value based on difference between Experiment and Control

/** Line Chart **/
GET Node/Edge simulation data
FOR Control run
plot concentration-over-time graph as blue curve
FOR Experiment run
plot Control concentration-over-time graph as blue curve
plot Experiment concentration-over-time graph as black curve
DISPLAY graph
```
